# The Lateral Membrane Organization and Dynamics of Myelin Proteins PLP and MBP Are Dictated by Distinct Galactolipids and the Extracellular Matrix

**DOI:** 10.1371/journal.pone.0101834

**Published:** 2014-07-08

**Authors:** Hande Ozgen, Waldemar Schrimpf, Jelle Hendrix, Jenny C. de Jonge, Don C. Lamb, Dick Hoekstra, Nicoletta Kahya, Wia Baron

**Affiliations:** 1 Department of Cell Biology, University of Groningen, University Medical Center Groningen, Groningen, The Netherlands; 2 Physical Chemistry, Department of Chemistry, Munich Center for Integrated Protein Science (CiPSM) and Center for Nanoscience (CeNS), Ludwig-Maximilians-Universität, Munich, Germany; Aix Marseille University, France

## Abstract

In the central nervous system, lipid-protein interactions are pivotal for myelin maintenance, as these interactions regulate protein transport to the myelin membrane as well as the molecular organization within the sheath. To improve our understanding of the fundamental properties of myelin, we focused here on the lateral membrane organization and dynamics of peripheral membrane protein 18.5-kDa myelin basic protein (MBP) and transmembrane protein proteolipid protein (PLP) as a function of the typical myelin lipids galactosylceramide (GalC), and sulfatide, and exogenous factors such as the extracellular matrix proteins laminin-2 and fibronectin, employing an oligodendrocyte cell line, selectively expressing the desired galactolipids. The dynamics of MBP were monitored by *z*-scan point fluorescence correlation spectroscopy (FCS) and raster image correlation spectroscopy (RICS), while PLP dynamics in living cells were investigated by circular scanning FCS. The data revealed that on an inert substrate the diffusion rate of 18.5-kDa MBP increased in GalC-expressing cells, while the diffusion coefficient of PLP was decreased in sulfatide-containing cells. Similarly, when cells were grown on myelination-promoting laminin-2, the lateral diffusion coefficient of PLP was decreased in sulfatide-containing cells. In contrast, PLP's diffusion rate increased substantially when these cells were grown on myelination-inhibiting fibronectin. Additional biochemical analyses revealed that the observed differences in lateral diffusion coefficients of both proteins can be explained by differences in their biophysical, i.e., galactolipid environment, specifically with regard to their association with lipid rafts. Given the persistence of pathological fibronectin aggregates in multiple sclerosis lesions, this fundamental insight into the nature and dynamics of lipid-protein interactions will be instrumental in developing myelin regenerative strategies.

## Introduction

Myelin is produced by oligodendrocytes (OLGs) in the central nervous system (CNS), where it enwraps axons in a multilamellar fashion to enable fast conduction of the action potential [1]. Myelin membranes contain a repertoire of myelin specific proteins of which proteolipid protein (PLP), a transmembrane protein, and the classical 18.5-kDa myelin basic protein (MBP) isoform, a peripheral membrane protein, are the most abundant ones. Compared to other plasma membranes, myelin has an unusual high lipid to protein ratio as 70% of its dry weight consists of lipids, including cholesterol and the galactolipids, galactosylceramide (GalC) and sulfatide [2]. These lipids often partition into so-called lipid rafts and the intercalation of a variety of myelin proteins in these membrane microdomains is pivotal for protein trafficking and myelin assembly [3]–[5]. For example, galactolipids play a prominent role in vesicle-mediated transport of PLP [3], [6], whereas a functional lateral role of the galactolipids in PLP's localisation in the plasma membrane is not known. Interestingly, as a cytoplasm-localized peripheral membrane protein, MBP does sense the presence of GalC and sulfatide, although both lipids localize at the extracellular surface of the myelin membrane [7]. Specifically, GalC and/or sulfatide containing liposomes induce a redistribution of GalC at the exoplasmic surface, via a so-called glycosynapse, thereby causing a concomitant redistribution of MBP at the cytoplasmic surface [8]–[10]. Additionally, the co-clustering of GalC together with MBP has been also observed when OLGs were treated with anti-GalC or anti-sulfatide antibodies [11], [12]. Moreover, the inhibition of GalC synthesis, and as a consequence that of sulfatide, causes a redistribution of MBP from raft to non-raft fractions [13]. Therefore, raft formation may be crucial for myelin assembly and a perturbation of the dynamic equilibrium of lipid rafts may result in an imbalance in the assembly of functional microdomains, leading to neurological disorders, such as multiple sclerosis (MS).

Apart from the important functional role of these structural myelin components for myelin assembly, a pivotal role of the surrounding extracellular matrix (ECM) is also apparent. ECM serves as a scaffold, providing signals to maintain and regulate cellular processes such as proliferation, migration and differentiation [14]. The ECM proteins fibronectin (Fn) and laminin-2 (Ln2) play opposite roles in the regulation of these cellular processes. Ln2, which harbors binding sites for sulfatide [14], [15], is known to promote myelin membrane formation [16]–[19], whereas Fn arrests intracellular vesicular transport in cultured OLGs and differentiation of oligodendrocyte progenitor cells to mature OLGs *in vivo* [3], [20]. Evidently, since the composition and function of the ECM is altered in neurological disorders including MS [21], [22], a detailed understanding of the specific role of ECM components in myelination is imperative, from both a fundamental and therapeutic point-of-view.

Lipid rafts, commonly characterized by their detergent insolubility, are considered dynamic membrane platforms, playing a role in various cellular processes [23], [24]. Biochemical isolation procedures of such membrane microdomains are technically challenging as the various experimental conditions may alter the results. Furthermore, although determination of the detergent (in)solubility of a specific membrane protein by biochemical means is very informative, by doing so, the dynamic information is obviously lost. Hence, biophysical techniques such as fluorescence correlation spectroscopy (FCS) [25]–[28] and raster image correlation spectroscopy (RICS) [29]–[31], which allow determination of the lateral diffusion of molecules in a non-invasive manner, are more appropriate if not ideal approaches for clarifying biological issues related to molecular dynamics. In this study, we applied, in conjunction with classical biochemical assays, different FCS techniques in living cells including *z*-scan point FCS, circular scanning FCS (s-FCS) and RICS to investigate the dynamics of membrane associated PLP and MBP in the presence of GalC and/or sulfatide, which were selectively expressed in oligodendrocyte-derived OLN-93 cells. In addition, we investigated whether Ln2 and Fn could modulate these dynamics and as such interfere with myelin biogenesis and assembly. The data revealed differences in the lateral diffusion coefficients of PLP and 18.5-kDa MBP, as dictated by their biophysical environment, specifically with regard to their association with detergent-resistant lipid rafts, and, in addition, by the nature of the ECM on which the cells were grown.

## Materials and Methods

### Cell culture and transfection

The rat derived oligodendrocyte progenitor cell line OLN-93 [32], a kind gift of Dr. Christiane Richter-Landsberg (University of Oldenburg, Germany), was cultured as described previously [33]. For the circular s-FCS, *z*-scan FCS and RICS measurements, cells were cultured on 8-well Labtek-I slides (VWR, Nunc, Naperville, IL), pre-coated with poly-L-lysine (PLL, 5 µg/ml, Sigma, St. Louis, MO), Fn (10 µg/ml, Sigma) and Ln2 (10 µg/ml, Sigma) in phenol-red free DMEM at a cell density 30,000 per well, one day prior to transfection. The cells were transfected with PLP-eGFP plasmid (pEGFP-N1-PLP, a kind gift of Dr. Niels Hellings, University of Hasselt, Belgium) or 18.5-kDa MBP-eGFP (pEGFP-C1-MBP-18.5-UTR, a kind gift of Dr. George Harauz, University of Guelph, Canada, [34]), using Lipofectamine™ 2000 Transfection Reagent (Invitrogen) as described in the manufacturer's instructions.

### Overexpression of galactolipids and thin layer chromatography (TLC)

The cDNAs encoding ceramide galactosyltransferase (cgt) and galactosylceramide 3′-sulfotransferase (cst) were kind gifts of Drs. Matthias Eckhardt (University of Bonn, Germany) and Brian Popko (University of Chicago, IL), respectively. cgt and cst were cloned into the *EcoRI* site of the retroviral pLXIN plasmid (Clontech Biosciences, Mountain View, CA). The production of retroviral particles and the subsequent infection and selection of OLN-93 cells were performed according to Maier et al. [35]. Briefly, OLN-93 cells were first transduced with cgt and subsequently selected for 10 days with 2 mg/ml geneticin to generate a polyclonal cell line that expresses GalC. To obtain a polyclonal cell line that expresses both GalC and sulfatide, this polyclonal cell line was subjected to a second transduction with cst. From the polyclonal cell lines, monoclonal OLN-G and OLN-GS cell lines were generated. To this end, the resistant cells were diluted to single isolated cells in 48 well plates, which were subjected to another selection procedure for 10 days. During the process of clone selection, we picked the clones that expressed GalC and/or sulfatide at their surface. OLN-mock cells were obtained by retroviral infection of OLN-93 cells with pLXIN (vector-only). The expression of GalC and/or sulfatide was characterized by TLC as described previously [36].

### Detergent extract preparation and OptiPrep density gradient centrifugation

One day after transfection with PLP-eGFP or 18.5-kDa MBP-eGFP, detergent extract preparation with 20 mM CHAPS and discontinuous OptiPrep density gradient centrifugation were performed as previously described [37]. Fractions were collected from top (fraction 1) to bottom (fraction 7). 250 µl was taken from each fraction and subjected to TCA precipitation [38] followed by Western blotting.

### Western Blot analysis

Samples were mixed with reducing sample buffer and heated for 30 min at 37°C. Proteins were separated by 10% SDS-PAGE and subjected to immunoblot analyses as described previously [33]. Primary antibodies used were polyclonal rabbit anti-GFP (1∶1000, Molecular Probes, Invitrogen), polyclonal rabbit anti-MBP (1∶1000, Dako Cytomation, Carpinteria, CA), polyclonal rabbit anti-caveolin-1 (1∶2000, Transduction Laboratories, Lexington, KY) and monoclonal mouse anti-Rho-GDI (1∶1000, Transduction Laboratories). IRDye®-conjugated were used as secondary antibodies (Li-Cor Biosciences, Lincoln, NE).

### Immunocytochemistry

24 hours after transfection with PLP-eGFP or 18.5-kDa MBP-eGFP, antibody staining of the cell surface lipids GalCer and sulfatide were performed on live cells at 4°C. After blocking non-specific binding with 4% bovine serum albumin in phosphate-buffered saline (PBS), cells were incubated with primary antibody for 30 min, washed three times and incubated for 25 min with TRITC-conjugated antibodies (Jackson ImmunoResearch, West Grove, PA). The cells were fixed with 4% paraformaldehyde (PFA) PBS for 20 min at RT, after which the nuclei were stained with DAPI (1 µg/ml, Sigma). O1 (anti-GalC) and O4 (anti-sulfatide) were both a kind gift of Dr. Guus Wolswijk [39]. Images were acquired by a confocal laser scanning microscope (Leica SP8 AOBS CLSM, Leica Microsystems, Heidelberg, Germany), equipped with an argon laser (488 nm), 2 He/Ne lasers (552 and 633 nm, respectively) and Leica Confocal Software. A 63×/1.25 oil immersion objective was used for 2-channel scanning (488 nm, 552 nm). Images of single cells were acquired with similar gain settings and 15 cells were measured at each condition. First, a stack of images was acquired to detect the best plane for analysis of the percentage co-localization. Afterwards, the co-localization coefficient was calculated by the Image-J plugin JACOPS as previously described [40]. After background subtraction, the optimal threshold value was defined separately for PLP-eGFP or 18.5-kDa MBP-eGFP and TRITC staining. The same threshold value was applied to all the images. The co-localization coefficient was calculated with the Manders Correlation Coefficient calculator. This analysis method gave rise to two correlation coefficients: the green pixels overlapping with the red channel (M1) or vice versa (M2). In order to calculate the percentage of co-localization at the plasma membrane, we used M2, which calculates overlapping red pixels (galactolipids) with green pixels (18.5-kDa MBP-eGFP or PLP-eGFP). In this manner, potential interference of the cytoplasmic signal that arises from free 18.5-kDa MBP-eGFP or PLP-eGFP in the cytoplasm was avoided. 100% co-localization gives a value of 1.

### Fluorescence fluctuation spectroscopy (FFS)

FCS and RICS measurements were performed on a home-built laser scanning pulsed interleaved excitation fluctuation imaging (PIE-FI) setup as described before [31], with the difference that a Nikon CFI Apo TIRF 100X Oil NA1.49 objective was used. Prior to the measurements, a calibration of the confocal volume was carried out by using a 5 nM Atto488-CA solution (D = 370 µm^2^/s at 22°C, diffusion coefficient application note of PicoQuant) with a total laser power of 10 µW before the objective (∼4 µW in solution). All *in vivo* measurements were performed at room temperature to reduce cell mobility and with an excitation power of 2 µW to minimize bleaching (∼0.8 µW in solution). The diffusion coefficients of both PLP-eGFP and 18.5-kDa MBP-eGFP were determined at the bottom plasma membrane to minimize distortion of the point-spread-function (PSF) of the oil objective. Moreover, in this manner, the effect of the different ECM proteins on the diffusion of the proteins could be studied. The software packages PAM and MIA [41] were used to analyze FCS and RICS data, respectively.

### z-scan FCS measurements

With a stack of images, we determined that 18.5-kDa MBP-eGFP had the highest fluorescence intensity near the bottom plasma membrane. In order to determine the optimal *z*-plane for each experiment, FCS measurements were performed at multiple *z*-positions. Hereby, the laser light was first focused slightly above the cell membrane and then subsequent point FCS measurements were performed at 10 different planes with a separation of 200 nm between planes, moving downwards (see Results). For *z*-positioning, a piezo stage (P-517.3CL; E-501-00, Physik Instrumente (PI) GmbH & Co. KG Karlsruhe, Germany) was used. To minimize axial drift and photobleaching during the measurement, the following scanning procedure was applied: all 10 planes were measured sequentially for 5 s. After completion of one stack, the first position was refocused using a home-built perfect focus system. The procedure was repeated a total of 12 times resulting in an acquisition time of 60 s per plane. From the *z*-position corresponding to the highest mean intensity (see Results), an autocorrelation was calculated and fitted using a 2D one component diffusion model with two exponential decays, describing a fast triplet (∼30 µs) and a slow eGFP blinking (∼500 µs):
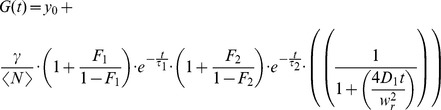



The fraction and decay time of the exponential decays are described by *F*_i_ and τ_i_ respectively. <N> and *D* correspond to the number and diffusion coefficient. The focal radius is given by *w*_r_ and *γ* is the geometry factor of a 2D Gaussian. Per condition, 7–10 cells were analyzed and fitted.

### z-scan RICS

The RICS method has been described in detail elsewhere [29]–[31]. Briefly, 24 hours after transfection with 18.5-kDa MBP-eGFP, cells were subjected to RICS measurements. As with point FCS measurements, a similar *z*-scan procedure was used. In total 10 planes were imaged 50 times each with 1-s acquisition time. The one corresponding to the highest fluorescence intensity was selected and analyzed for each cell.

### Circular scanning FCS measurements (s-FCS)

To determine the diffusion coefficient of PLP-eGFP, FCS experiments were performed in cells while scanning the laser focus in *x-y* over the membrane in a 4.5-µm-diameter circle at an orbit frequency of 5 kHz over 6 minutes and a laser power of 2 µW before the objective. No significant photobleaching was observed during data acquisition. The collected data was divided into 200 bins per scanning orbit, corresponding to the different laser positions. Within a bin the laser focus was considered stationary (laser displacement only approximately 70 nm). For each bin, an intensity trace and auto-correlation function (ACF) was calculated, and both were averaged over 10 consecutive positions to increase signal-to-noise of the data. Then, points with clearly aberrant intensity or ACF traces, likely caused by vesicles diffusing through the focus, were omitted and all remaining bins of several measurements per cell were averaged (see Results). In this way, diffusion coefficients of PLP could be measured, even in the presence of vesicular movement. Given the manual selection of outliers, albeit with direct feedback from the correlation, the actual diffusion coefficients might slightly differ. GPI-anchored GFP was used as a control to verify whether the method worked satisfactorily (data not shown). The average ACFs of different independent experiments were fitted globally with the following 2D anomalous diffusion model;
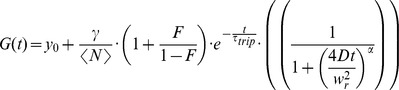



Where *γ* is the geometry factor for the 2D Gaussian and *y*_0_ is the offset. <*N*> is the average number of molecules in the observation volume. *F* and *τ*_trip_ are the dark state fraction and the blinking time, respectively, accounting for the slow blinking behavior of eGFP [42]. *D* represents the diffusion coefficient, *w*_r_ the radius of the focus and *α* the anomaly coefficient.

### Statistical analysis

All data are represented as the mean ± standard error of the mean (SEM) of at least three independent experiments. The statistical significance was calculated by a two-tailed Student′s t-test for comparison between two means and by a one-way ANOVA followed by Newman-Keuls posttest to compare more than two means. A p value of p<0.05 was considered statistically significant.

## Results

### 18.5-kDa MBP associates with CHAPS-insoluble microdomains in GalC-expressing OLN-93 cells

**Figure 1:**
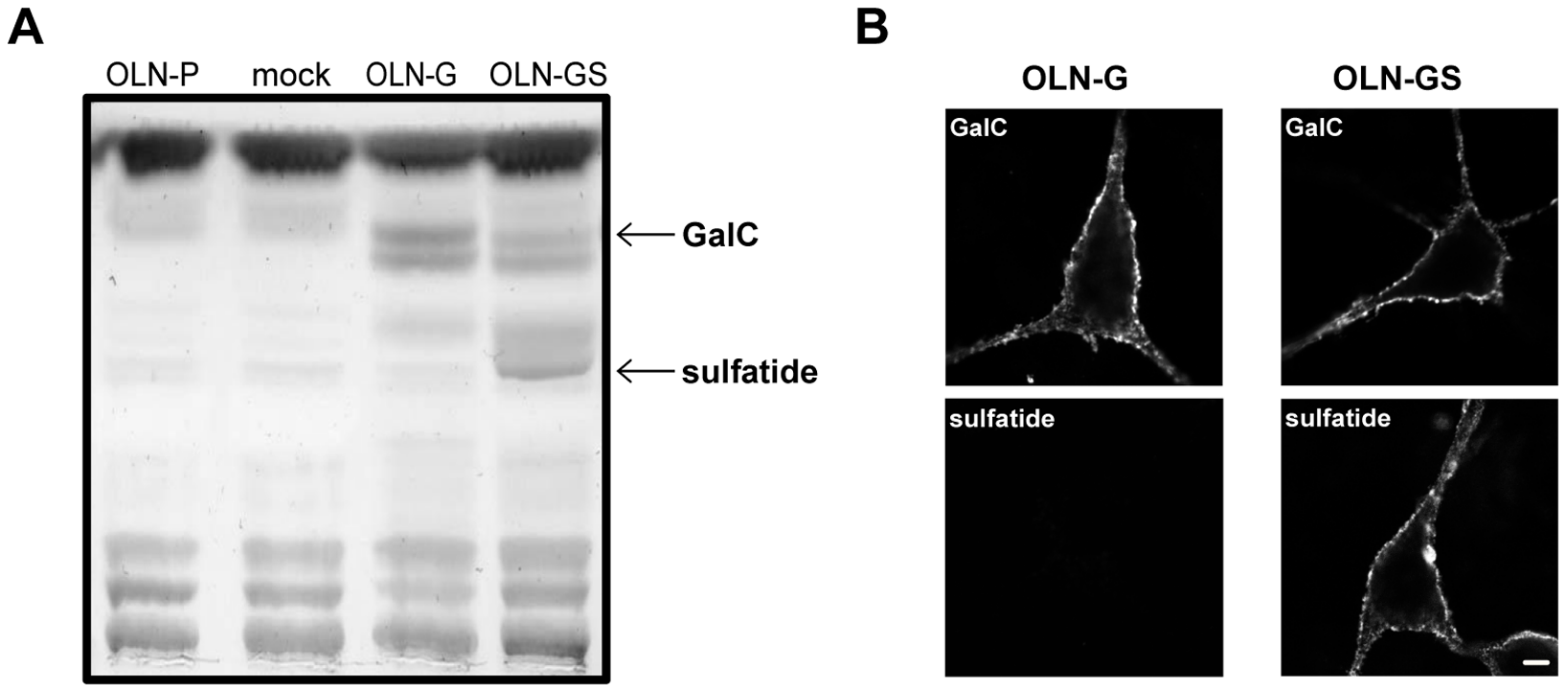
Expression and localization of galactolipids in OLN-93 cells. A) The expression of GalC and sulfatide was characterized in OLN-P, OLN-G, OLN-GS and mock-transduced cells by TLC. **B**) OLN-G and OLN-GS cells were cultured on PLL and stained for cell surface GalC or sulfatide with O1 and O4 antibodies, respectively. Scale bar is 5 µm.

To investigate the effect of the major myelin galactolipids GalC and sulfatide on the dynamics and lateral membrane organization of the myelin-specific proteins MBP and PLP, we took advantage of a rat derived oligodendrocyte cell line, OLN-93. These cells represent immature OLGs and they neither synthesize PLP and MBP nor GalC and sulfatide. Accordingly, this enabled us to apply a bottom-up approach where we first stably overexpressed the enzymes (see Materials and Methods) responsible for the synthesis of GalC and sulfatide, respectively, and then transiently introduced 18.5-kDa MBP or PLP. As a result, in addition to OLN-93 parental (OLN-P) cells devoid of either galactolipid, we generated OLN-93 monoclonal cell lines expressing only GalC (OLN-G) and OLN-93 cells expressing both GalC and sulfatide (OLN-GS). To verify the presence of these lipids, total lipid extracts were analyzed by TLC. Whereas OLN-P cells and OLN-93 cells transduced with vector-only (mock) were negative for both GalC and sulfatide, GalC was present in OLN-G and OLN-GS cells, while sulfatide was only detectable in OLN-GS cells (Figure 1A). Examination of the galactolipid-expressing cells by live staining fluorescence microscopy revealed that both lipids localized at the plasma membrane of the cells and display a heterogeneous, patchy distribution (Figure 1B), similar to what is seen in primary oligodendrocytes [43].

**Figure 2:**
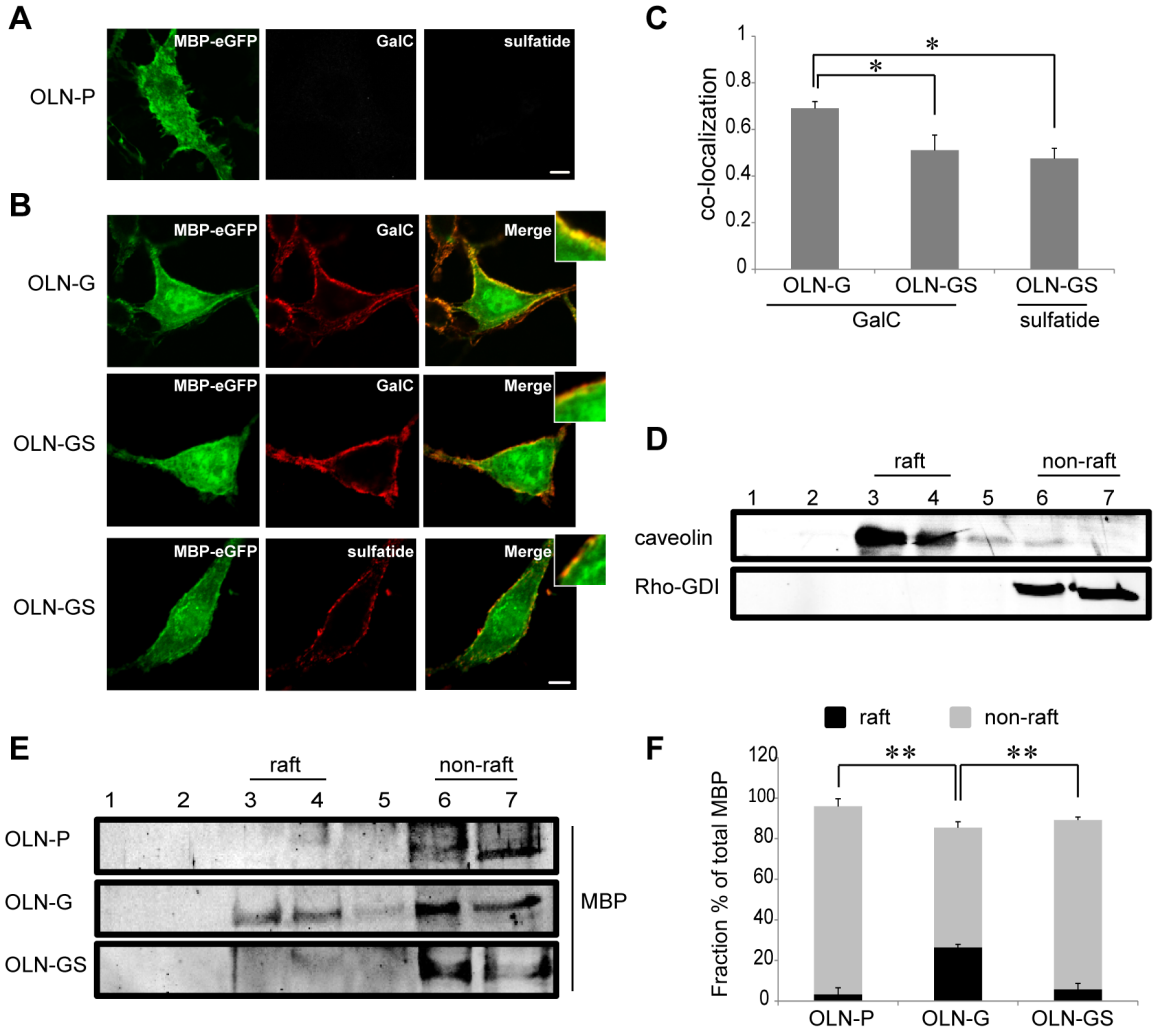
Co-localization of MBP with galactolipids at the plasma membrane of OLN-93 cells and its microdomain association. OLN-P, OLN-G and OLN-GS cells were cultured on PLL and transiently transfected with 18.5-kDa MBP-eGFP. The experiments were performed 24 hours after transfection. **A,B**) Representative images of transfected cells stained for either cell surface GalC or sulfatide with O1 and O4 antibodies (red), respectively. Scale bar is 6 µm. Insets show higher power magnifications of which brightness-contrast and median filtering were performed by Image J. **C**) The fractional co-localization between detection channels was determined by the Manders Correlation Coefficient Calculator (see Materials and Methods) and displayed as a bar graph. Bars represent the mean+SEM of at least 15 cells. The statistical analysis was performed by GraphPad Prism 5 (one-way ANOVA followed by the Newman-Keuls posttest, * p<0.05). **D–F**) OptiPrep density gradient centrifugation analysis after CHAPS extraction. Caveolin-1 and Rho-GDI are a positive and negative marker for membrane microdomains (lipid rafts), respectively (**D**). 18.5-kDa MBP-eGFP expression was determined using an anti-MBP polyclonal antibody (**E**). Representative Western blots are shown. The intensities of the MBP-eGFP bands in **E** were quantified by Image J. The total protein expression was calculated by adding the intensity of all the fractions. The protein percentage of each fraction was then calculated by dividing the protein intensity present in that fraction by total protein expression. A bar graph of the distribution of MBP-eGFP is shown. The fraction percentage of (raft) fractions 3 and 4 are plotted in black and the fraction percentage of (non-raft) fractions 6 and 7 are shown as grey bars. Bars represent the mean+SEM. The statistical analysis was performed using GraphPad Prism 5 (n = 3, one-way ANOVA followed by the Newman-Keuls posttest, ** p<0.01).

After having determined the expression and surface localization of GalC and sulfatide in the OLN-G and OLN-GS monoclonal cell lines, we transiently transfected the cells with eGFP-tagged 18.5-kDa MBP. To solely determine the effect of extracellular leaflet localized galactolipids on the intracellular distribution of inner leaflet associated peripheral membrane protein 18.5-kDa MBP, we first examined the effect of OLN-P, OLN-G and OLN-GS on the inert coating material PLL. As shown in Figure 2A and B, 18.5-kDa MBP distributed diffusely throughout the cytoplasm in all cell types revealing an occasional patchy appearance particularly at or near the plasma membrane. Next, we further characterized the distribution of 18.5-kDa MBP at the plasma membrane in the context of cellular surface expression of GalC and sulfatide. Interestingly, following surface staining of GalC and sulfatide in OLN-G and OLN-GS cells, a substantial co-labeling of 18.5-kDa MBP and GalC was observed in OLN-G cells, which was less in cells expressing both galactolipids. To obtain further support for these findings, a quantitative co-localization percentage was calculated using the Manders Correlation Coefficient Calculator, where 100% co-localization is represented by 1 (see Materials and Methods). As shown in Figure 2C, the fractional co-distribution of 18.5-kDa MBP with GalC was indeed significantly higher in OLN-G than in OLN-GS cells (0.69±0.03 and 0.51±0.06 respectively), while a similar fractional co-distribution as GalC was obtained with sulfatide in OLN-GS cells (0.47±0.04). These data suggest that 18.5-kDa MBP, being a peripheral membrane protein and interacting with the cytoplasmic leaflet of the myelin membrane, senses the presence of the galactolipids in the outer leaflet, in particular GalC, assuming that glycosphinglipids are exclusively present in the outer leaflet of the plasma membrane [8]–[10].

In different cell types, including OLGs, GalC and sulfatide are able to integrate into small membrane microdomains known as lipid rafts or detergent-resistant membranes [4], [23], [44]. It is important to note that the co-localization analysis does not necessarily imply that MBP and galactolipids directly interact; rather the data reveal that galactolipids and MBP might localize within the same membrane domain. Within this context, the data would indicate that MBP prefers integration within GalC-enriched microdomains. MBP is known to be incorporated in CHAPS-resistant microdomains [13], [45]. To determine whether the presence of either or both galactolipids affected the membrane microdomain association of 18.5-kDa MBP, the various cell lines were extracted with CHAPS and analyzed using OptiPrep gradient analysis. In the gradients, fractions 3 and 4 are considered to represent insoluble-raft fractions, as reflected by the localization of the well-established raft marker caveolin-1, whereas fractions 6 and 7 are considered to represent soluble-non-raft fractions in which the negative control Rho-GDI localizes (Figure 2D). As shown in Figure 2E and F, only minor amounts of 18.5-kDa MBP, if at all, were detected in raft fractions for both control OLN-P cells and OLN-GS cells. Specifically, 92.7±6.5% (OLN-P) and 83.4±2.5% (OLN-GS) of the extracted protein fractions were soluble in CHAPS. In contrast, a substantial fraction of the 18.5-kDa MBP pool (26.5±2.5%) expressed in the OLN-G cells (Figure 2D and E) localized in CHAPS-insoluble raft fractions, implying that in this case GalC represents the driving force for MBP′s (partial) association with CHAPS-insoluble microdomains. Of note, an increase in CHAPS-resistance of 18.5-kDa MBP was also observed in another OLN-G monoclonal cell line, and not in another OLN-GS monoclonal cell line (data not shown).

### The lateral mobility of 18.5-kDa MBP is increased in GalC-expressing OLN-93 cells

**Figure 3:**
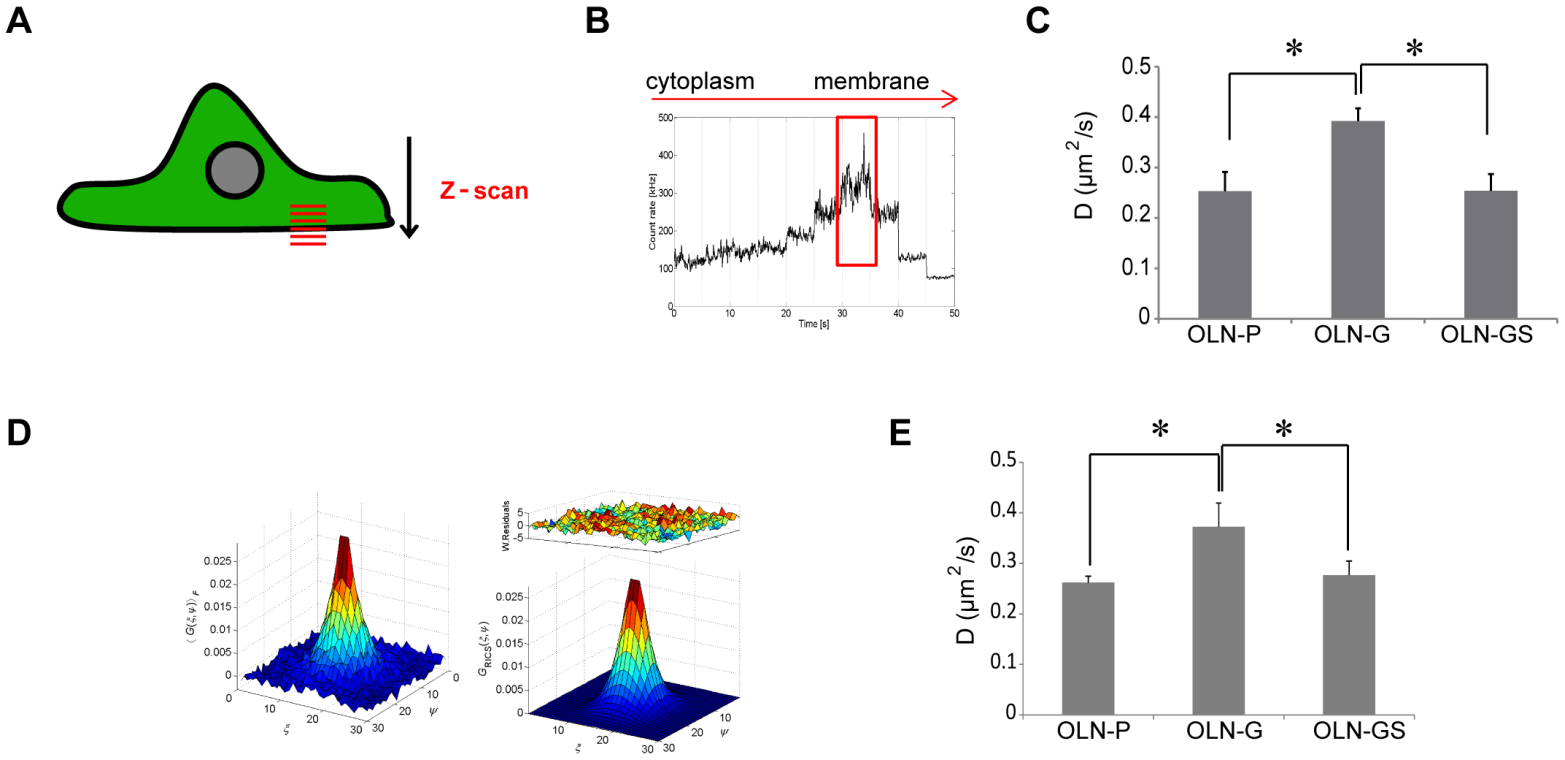
Lateral mobility of 18.5-kDa MBP in OLN-93 cells. *z*-scan FCS and *z*-scan RICS measurements were performed on OLN-P, OLN-G and OLN-GS cells cultured on PLL and transiently transfected with 18.5-kDa MBP-eGFP. Experiments were performed 24 hours after transfection. **A**) A schematic of a cell showing *z*-scanning at the basal plasma membrane. **B**) The total intensity as a function of *z*-position for a typical z-scanning measurement is shown. **C**) The averaged autocorrelation curves from 10 cells were fitted with a 2D one component diffusion model. The diffusion coefficients from *z*-scan FCS are shown as a bar graph. **D**) A representative autocorrelation curve and corresponding 2D1C fit model is shown from a *z*-scan RICS measurement at the ventral plasma membrane. **E**) The diffusion coefficients for the *z*-scan RICS experiments are shown as a bar graph and represent the average of at least 10 cell measurements. Bars (**C,E**) represent the mean+SEM. The statistical analysis was performed using GraphPad Prism 5 (one-way ANOVA followed by the Newman-Keuls posttest, * p<0.05).

To verify whether the galactolipid-dependent differences in raft partitioning and/or apparent co-distribution of 18.5-kDa MBP were also reflected by differences in its lateral mobility, we performed single-point FCS measurements. A *z*-stack of images was collected to determine the position of the bottom plasma membrane where the maximum amount of protein was localized (illustrated in Figure 3A). Specifically, the measurements were performed in 10 *z*-planes, each 200 nm apart, starting in the middle of the cell and gradually moving down. FCS was performed in the plane with the highest fluorescence intensity of 18.5-kDa MBP (Figure 3B), and the FCS curves were averaged and fitted empirically with a 2D one component diffusion model. Our results show that the diffusion rate of 18.5-kDa MBP in OLN-G cells is significantly higher (0.39±0.03 µm^2^/s) than in OLN-P cells (0.25±0.04 µm^2^/s) and OLN-GS cells (0.25±0.03 µm^2^/s) (Figure 3C). To obtain further support for these observations, we also applied RICS to determine the lateral diffusion rate of 18.5-kDa MBP in control and galactolipid-expressing cells. For similar reasons as for FCS, we also combined *z*-scan with RICS, keeping the same settings. Following the measurements, the data was fitted with a one component model (Figure 3D). Consistent with *z*-scan FCS, we observed that the diffusion rate of 18.5-kDa MBP in OLN-G cells was significantly higher (0.37±0.04 µm^2^/s) than the rates observed in OLN-P cells (0.23±0.01 µm^2^/s) and OLN-GS cells (0.28±0.03 µm^2^/s) (Figure 3E).

### PLP preferentially associates with CHAPS-insoluble microdomains in GalC- and sulfatide-overexpressing OLN-93 cells

**Figure 4:**
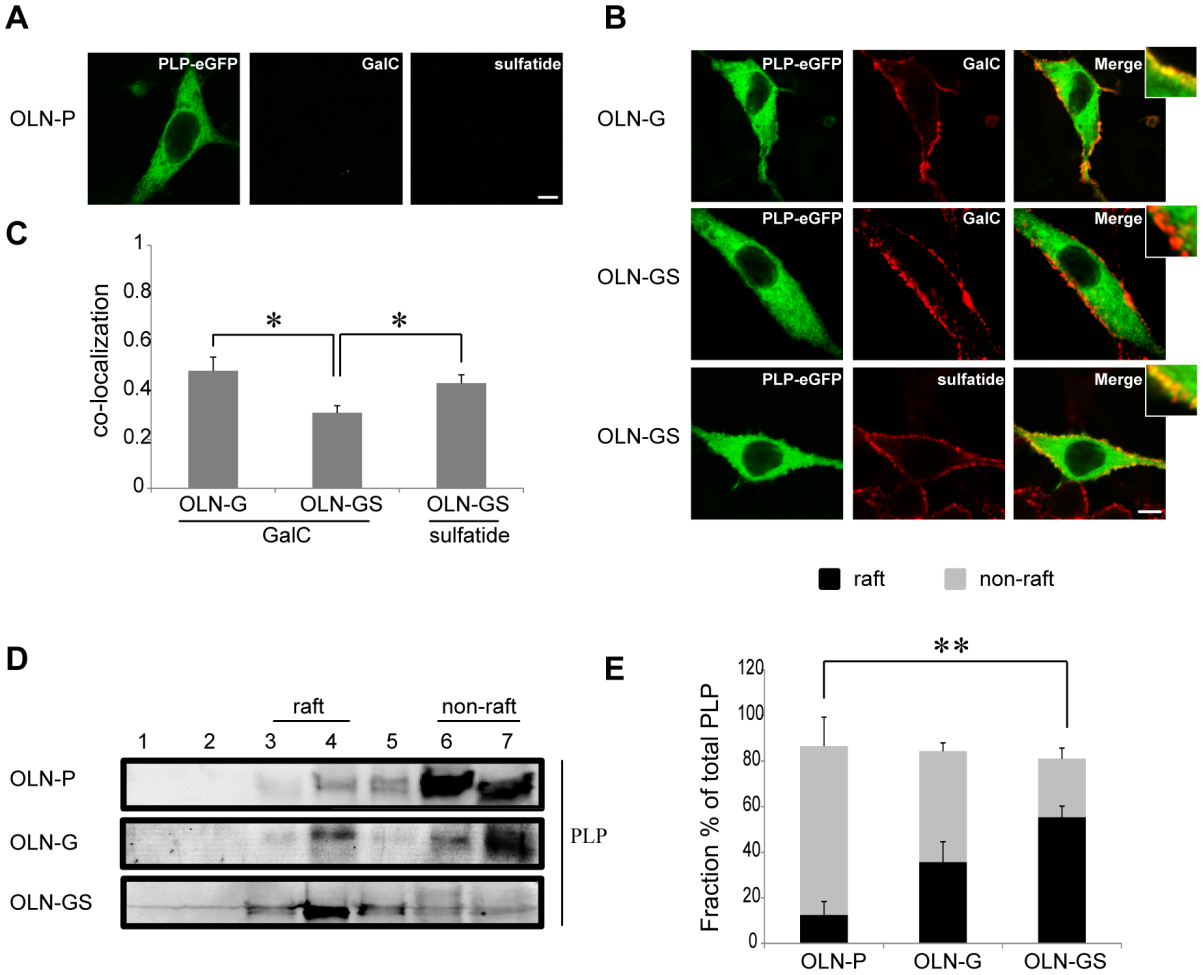
Co-localization of galactolipids with PLP at the plasma membrane of OLN-93 cells and its microdomain association. OLN-P, OLN-G and OLN-GS cells were cultured on PLL and transiently transfected with 18.5-kDa MBP-eGFP. The experiments were performed 24 hours after transfection. **A,B**) Representative images of transfected cells stained for either cell surface GalC or sulfatide with O1 and O4 antibodies (red), respectively. Scale bar is 6 µm. Insets show higher power magnifications of which brightness-contrast and median filtering were performed by Image J. **C**) The fractional co-localization between detection channels was determined by the Manders Correlation Coefficient Calculator (see Materials and Methods) and displayed as a bar graph. Bars represent the mean+SEM of at least 15 cells. The statistical analysis was performed using GraphPad Prism 5 (one-way ANOVA followed by the Newman-Keuls posttest, *p<0.05). **D,E**) OptiPrep density gradient centrifugation analysis after CHAPS extraction. PLP-eGFP expression was determined using an anti-GFP polyclonal antibody (**D**). Representative Western blots are shown. The percentages of the PLP-eGFP bands were quantified as described in the legend to Figure 2 for MBP-eGFP bands (**E**). A bar of the distribution of PLP-eGFP is shown. The raft fractions are plotted in black and the non-raft fractions are shown as grey bars. Bars represent the mean+SEM. The statistical analysis was performed using GraphPad Prism 5 (n = 3, one-way ANOVA followed by the Newman-Keuls posttest, **p<0.01).

Next, it was of particular interest, given the uniqueness of the OLN-93 cell system, to investigate whether GalC and/or sulfatide could affect the lateral distribution and dynamics of the integral membrane protein PLP. To obtain detailed information on subcellular localization in a reliable manner, *z*-stacks were taken and the three-dimensional information analyzed. As shown in Figure 4A and B, besides localization in the plasma membrane, PLP-eGFP was found predominantly in intracellular vesicular structures and distributed throughout the cytoplasm, which is comparable and consistent with that seen in primary rat OLG mono-cultures [38]. To investigate a potential galactolipid-dependent effect on this distribution, the OLN-93 cells were subsequently subjected to surface galactolipid immunostaining. Interestingly, prominent co-labeling of PLP and GalC appear to occur in OLN-G cells, whereas very little co-localization between GalC and PLP was apparent in cells expressing both GalC and sulfatide (OLN-GS) (Figure 4B). Remarkably, in cells expressing both galactolipids, PLP showed a most pronounced and preferential co-localization with sulfatide (Figure 4B, lower panel), rather than GalC. The co-distribution percentage as obtained with the Manders Correlation Coefficient Calculator confirmed these observations. Thus, in OLN-G cells, which are devoid of sulfatide, a degree of co-localization of GalC and PLP was observed that amounted up to 0.48±0.06 (Figure 4C). This level of co-localization of GalC and PLP decreased considerably in the OLN-GS cells (0.31±0.03; Figure 4C). Indeed, in OLN-GS cells, PLP preferentially co-localized with sulfatide (0.43±0.03; Figure 4C). PLP, like MBP, displays a CHAPS detergent insolubility in myelin membranes [3], [36]. To investigate whether GalC and sulfatide similarly affect this biochemical property of PLP in OLN-93 cells, we determined the protein′s detergent (in)solubility in parental and galactolipid-expressing OLN-93 cells by CHAPS extraction followed by OptiPrep density gradient analysis. As shown in Figure 4D and E, while in parental OLN-P cells the majority of PLP (74.1±22.1%) resided in CHAPS-soluble fractions, in OLN-GS the majority of the protein localized in CHAPS-insoluble fractions (55.4±8.1%). In GalC-expressing cells, we observed an intermediate distribution where PLP resided in both CHAPS-soluble and insoluble fractions. Similar findings were observed in another OLN-G and OLN-GS monoclonal cell line, while sodium chorate-mediated inhibition of sulfatide expression in OLN-GS cells counteracted the CHAPS-resistance of PLP (data not shown). Together, these data suggest that PLP preferentially associates with sulfatide and co-localizes with this lipid in membrane microdomains, characterized by their insolubility in CHAPS.

### The lateral mobility of PLP is decreased in GalC and sulfatide expressing OLN-93 cells

**Figure 5:**
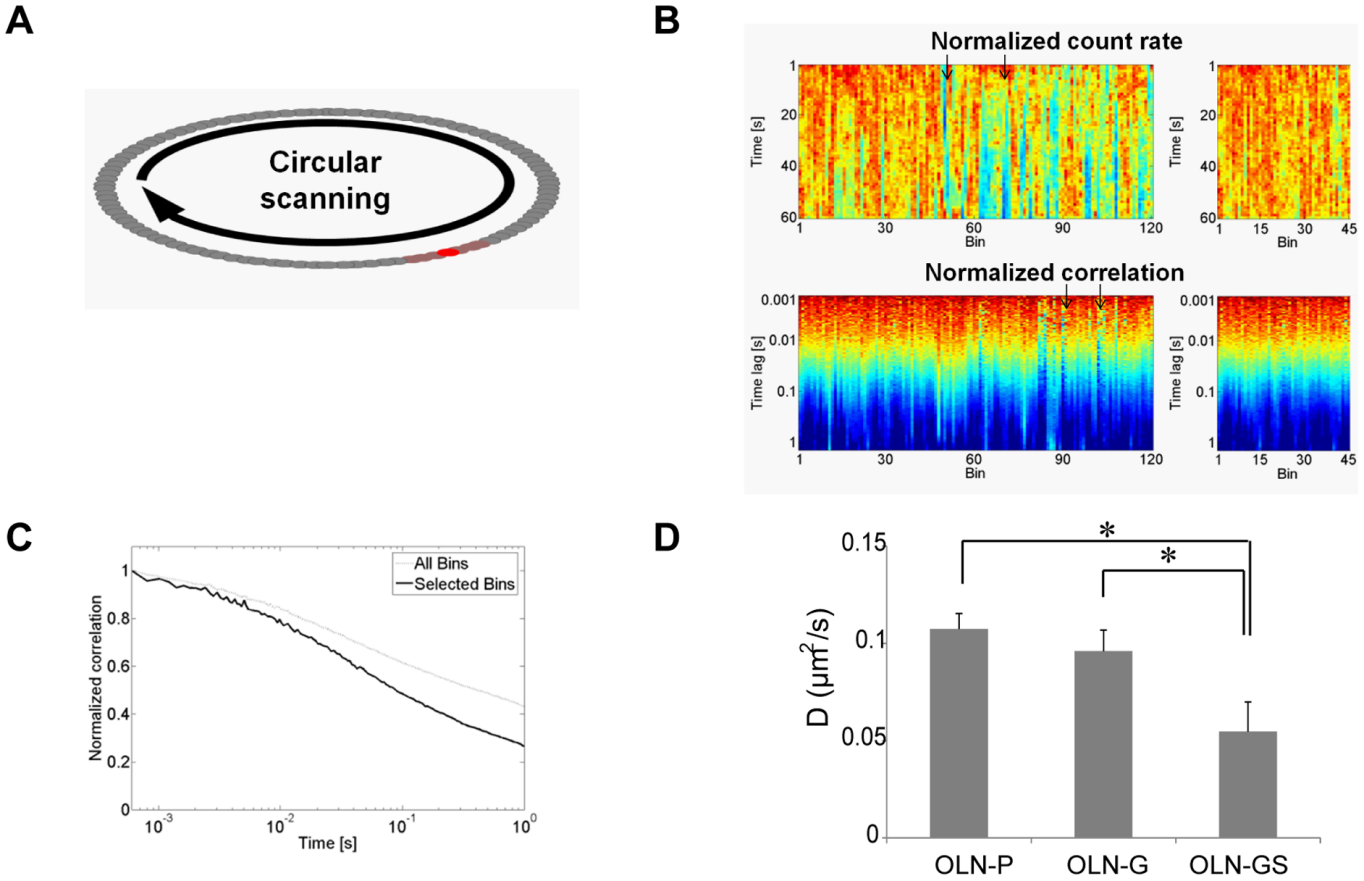
Lateral mobility of PLP in the presence of GalC and sulfatide as determined by s-FCS. OLN-P, OLN-G and OLN-GS cells were transiently transfected with PLP-eGFP on PLL and 24 hours after transfection the mobility was measured using circular scanning-FCS. **A**) The scanned circle was divided into bins and each individual bin (e.g. highlighted by a red spot) was correlated. For analysis, 10 bins were averaged (dark red area). **B**) An averaged intensity and correlation carpet for PLP-eGFP diffusion in an OLN-P cell are shown. Inhomogeneous intensity traces were discarded and the remaining FCS curves averaged (arrows). **C**) The autocorrelation function for all bins (dotted line) and selected bins (black line) where intensity heterogeneities that disturb the measurements have been removed. **D**) The diffusion coefficients are presented as a bar graph. The bars represent mean+SEM of at least 7 cell measurements. The statistical analysis was performed using GraphPad Prism 5 (one-way ANOVA followed by the Newman-Keuls posttest *p<0.05).

To examine whether the galactolipid-dependent differences in PLP′s biophysical environment might be reflected by differences in its dynamics, we determined the lateral mobility of PLP-eGFP in OLN-P, OLN-G and OLN-GS cells. From a technical perspective, it was not possible to monitor the dynamics of PLP by single point FCS neither by RICS, as PLP is highly present in bright and mobile cytoplasmic vesicles (Figure 4A, [38]) that interfere with the data acquisition and analysis. Therefore, to investigate PLP′s dynamics at the bottom plasma membrane in living cells, we applied circular s-FCS, a technique previously used to measure dynamics of proteins and lipids in giant unilamellar vesicles (GUVs; [28], [46]) and living cells [47], and combined this with intensity carpet analysis. In order to collect the signal coming exclusively from the plasma membrane and not from the vesicles present in the cytoplasm, we used a high numerical aperture objective. Although the laser focus was on the plasma membrane, the confocal PSF extended about a micrometer into the cytoplasm. Since the vesicles were highly mobile and constituted very bright particles, the resulting correlations were heavily biased towards this vesicular movement. In order to overcome this limitation, the data was not recorded from a single point, but rather by data collection while scanning a circle, as illustrated in Figure 5A, and further processed as described in Materials and Methods section. Both intensity traces and FCS curves were plotted as carpets where the x-axis represented the bins and the y-axis the time (Figure 5B). Lines that show a very heterogeneous intensity trace were likely caused by a vesicle diffusing through the focus (Figure 5B, arrows). Using the individual point correlation functions as direct feedback, we manually removed these outliers in a straightforward manner and thus minimize the influence of the movement of cellular compartments. The remaining FCS curves of such measurements were then averaged, giving rise to a single FCS curve per cell, and fitted with an anomalous diffusion model (Figure 5C). The fit revealed that the lateral diffusion coefficient of PLP in the OLN-93 plasma membrane was substantially slower in sulfatide-expressing OLN-GS cells (0.06±0.02 µm^2^/s; α = 0.467) than in GalC-expressing OLN-G cells (0.10±0.01 µm^2^/s; α = 0.529) or parental OLN-P cells (0.11±0.01 µm^2^/s; α = 0.569) (Figure 5D). Accordingly, these data indicate that the preferential association of PLP with sulfatide-enriched domains and their co-localization in CHAPS-resistant fractions, or CHAPS-domains, is reflected by a pronounced decrease in the protein′s lateral mobility in OLN-GS cells.

### Fibronectin prevents co-localization of PLP and sulfatide

**Figure 6:**
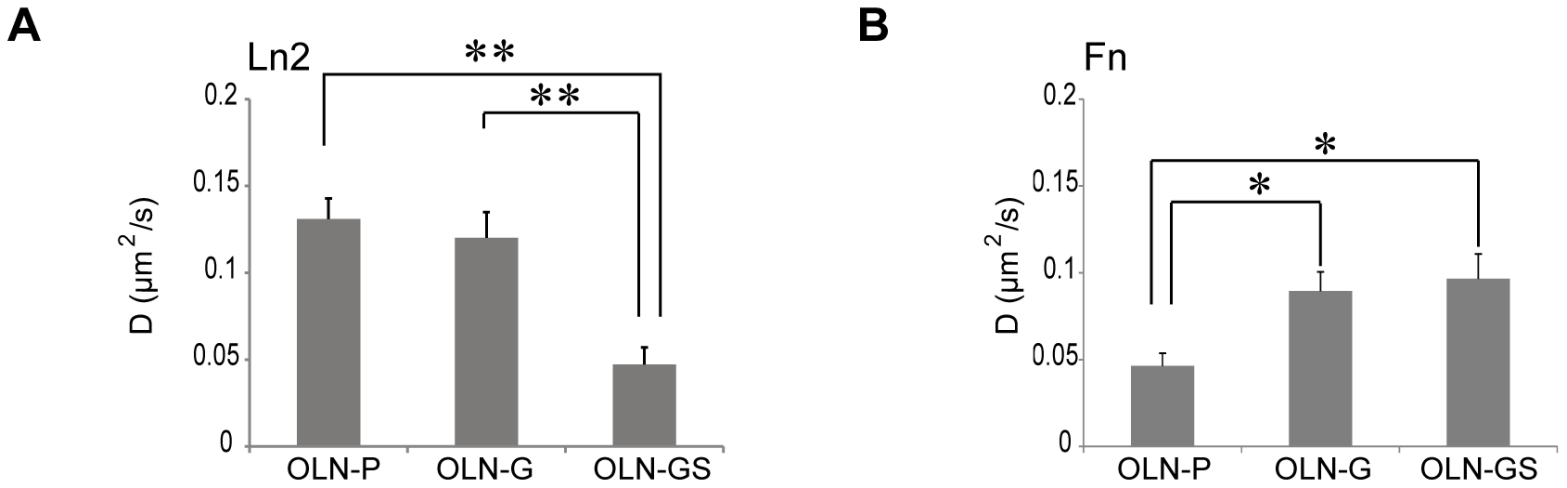
Lateral mobility of PLP in the presence of GalC and sulfatide on Ln2 and Fn. **A,B**) s-FCS experiments were performed on OLN-P, OLN-G and OLN-GS cells that were plated on either Ln2 or Fn and transiently transfected with PLP-eGFP. The experiments were performed 24 hours after transfection. The averaged autocorrelation curves from at least 7 cells were fitted with an anomalous diffusion model. The diffusion coefficients of the slow component (i.e., the diffusion term) are presented as a bar graph (Ln2, **A** and Fn, **B**). The data were plotted as mean+SEM and statistical analysis was performed using GraphPad Prism 5 (one-way ANOVA followed by the Newman-Keuls posttest, * p<0.05, ** p<0.01).

**Figure 7:**
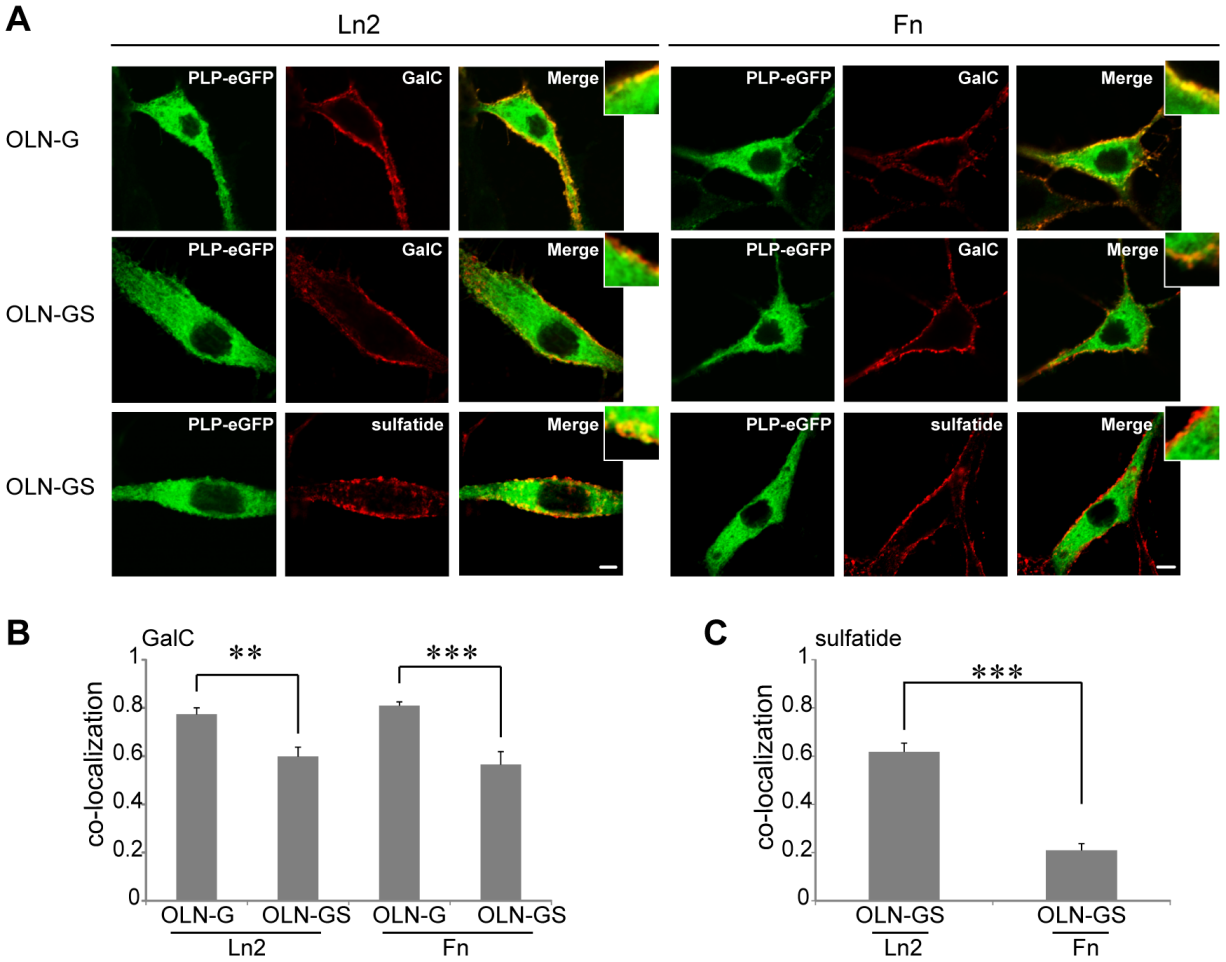
Co-localization of galactolipids with PLP at the plasma membrane of OLN-93 cells on Ln2 and Fn. **A**) OLN-G and OLN-GS cells were cultured on either Ln2 (left panel) or Fn (right panel), transiently transfected with PLP-eGFP, and 24 hours after transfection stained for either cell surface GalC or sulfatide with O1 and O4 antibodies (red), respectively. Representative images are shown. Scale bar is 6 µm. Insets show higher power magnifications of which brightness-contrast and median filtering were performed by Image J. **B,C**) The fractional co-localization between detection channels was determined by the Manders Correlation Coefficient (see Materials and Methods) and displayed as a bar graph. Bars represent the mean+SEM of at least 15 cells. The statistical analysis was performed using GraphPad Prism 5 [student′s t-test, ** p<0.01, *** p<0.001 (OLN-G and OLN-GS on the same ECM substrate)].

As we measure lateral mobility of 18.5-kDa MBP-eGFP and PLP-eGFP at the bottom plasma membrane, it was next of particular interest to analyze its dynamics as a function of two myelination affecting ECM components, i.e., Fn and Ln2, of which we and others have shown previously that they either strongly inhibit or promote myelination, respectively [18], [20], [48]. To investigate the potential ability of ECM proteins Ln2 and Fn to interfere with myelin biogenesis and assembly, we first evaluated the lateral membrane dynamics of 18.5-kDa MBP and PLP in OLN-93 cells cultured on either ECM substrate. *z*-scan FCS analysis revealed that no significant differences in the diffusion rates of 18.5-kDa MBP were detected for the different cell types on the different ECM substrates (data not shown). s-FCS measurements showed that the lateral diffusion rate of PLP in OLN-93 cells grown on Ln2 was similar in parental cells (OLN-P) and in GalC-expressing cells, i.e., 0.13±0.01 µm^2^/s and 0.12±0.02 µm^2^/s, respectively. However, in the case of OLN-GS cells grown on Ln2, the rate decreased to 0.05±0.01 µm^2^/s (Figure 6A), indicating that the diffusion coefficient of PLP was significantly slower when the membrane also contained sulfatide, similar as observed when cells were grown on PLL (Figure 5D). In marked contrast, when grown on Fn, the galactolipid expressing cells showed a considerably faster diffusion coefficient of PLP. In OLN-G cells and OLN-GS cells, a diffusion coefficient of 0.09±0.01 µm^2^/s and 0.11±0.01 µm^2^/s were determined respectively, whereas in OLN-P cells only a lateral diffusion coefficient of 0.05±0.01 µm^2^/s was obtained (Figure 6B). Remarkably, when comparing the diffusion coefficients of PLP in OLN-G cells grown either on PLL (0.10±0.01 µm^2^/s), Fn (0.09±0.01 µm^2^/s) or Ln2 (0.12±0.02 µm^2^/s), no significant differences were observed, which further supports the specificity of sulfatide in ECM-dependent PLP processing. Given the observed quantitative differences in lateral diffusion rates of PLP in the presence of different galactolipids as a function of the nature of the ECM, we then further investigated the co-localization degree of PLP with GalC and sulfatide on Ln2 and Fn. When grown on Ln2, the degree of co-localization of PLP with GalC in OLN-G cells (0.77±0.03) was significantly higher than the level of co-localization observed with GalC and sulfatide in OLN-GS cells (0.59±0.04 and 0.62±0.04 respectively, Figure 7A and B). A similar analysis of cells grown on Fn revealed that the degree of co-localization of PLP with GalC was also in this case significantly higher in OLN-G cells than in OLN-GS cells (0.81±0.02 and 0.57±0.05, respectively; Figure. 7A and B). Notably, on both ECM substrates the degree of co-localization of PLP and GalC was higher as compared to PLL. In contrast, we observed very little if any co-localization of PLP and sulfatide in OLN-GS cultured on Fn (0.21±0.03; Figure 7A and C). Compared to the value of 0.62±0.04, as obtained for the co-localization of PLP and sulfatide on Ln2, these data imply that Fn abolished the partitioning of PLP in sulfatide-enriched domains, without significantly affecting PLP′s integrations within GalC-domains.

### PLP is recruited into sulfatide-enriched microdomains in an ECM-dependent manner

**Figure 8:**
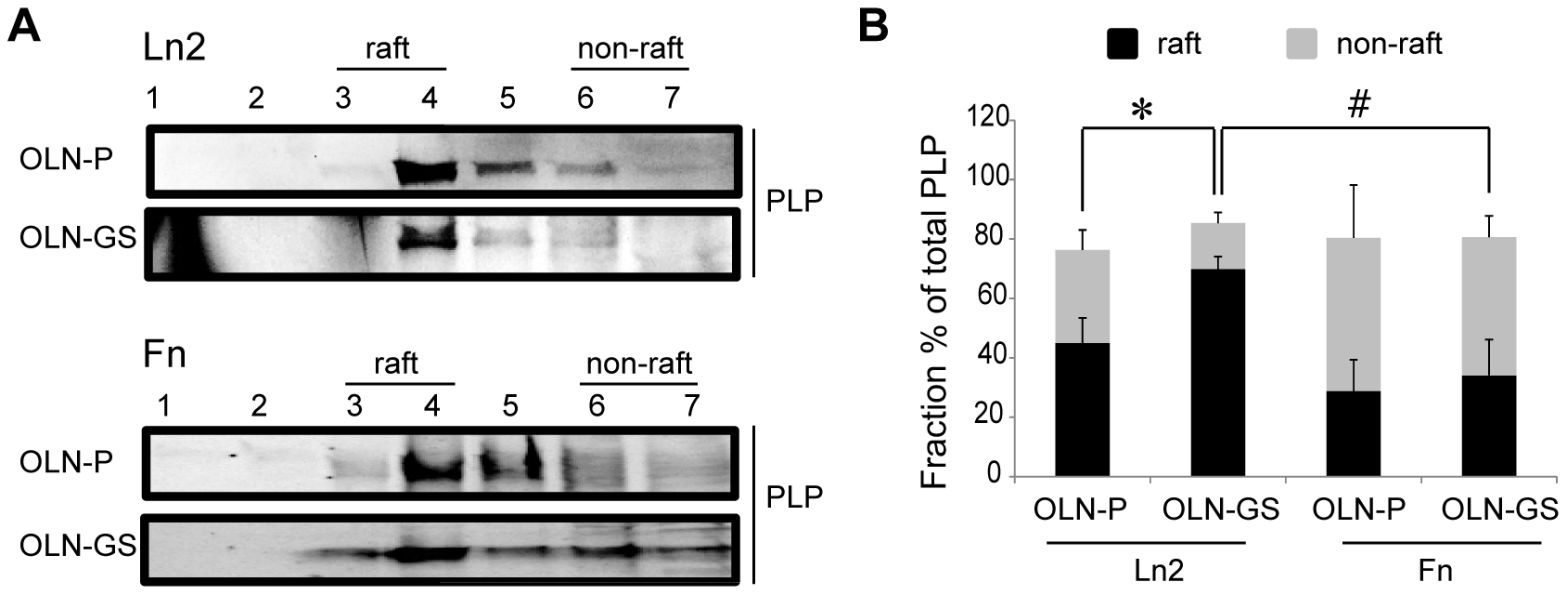
Microdomain association of PLP on the ECM proteins Ln2 and Fn. OptiPrep density gradient centrifugation experiments were performed on OLN-P and OLN-GS cells that were plated on either Ln2 or Fn and transiently transfected with PLP-eGFP. Experiments were performed 24 hours after transfection. PLP-eGFP expression was determined using an anti-GFP polyclonal antibody Representative Western blots are shown (**A**) The percentages of the PLP-eGFP bands were quantified as described in the legend to Figure 2. A bar graph of the distribution of PLP-eGFP is shown (**B**). The raft fractions are plotted in black and the non-raft fractions are shown as grey bars. The bars represent the mean+SEM. The statistical analysis was performed using GraphPad Prism 5 [n = 3, student′s t-test, * p<0.05 (OLN-P and OLN-GS on the same ECM substrate), # p<0.05 (OLN-GS or OLN-P on Fn and Ln2)].

Significant differences in the lateral diffusion coefficients and co-localization degrees of PLP were observed in OLN-P cells and OLN–GS cells dependent on the composition of the ECM. To rationalize these intriguing differences, we took into account that a shift of PLP from a CHAPS-soluble to CHAPS-insoluble fraction was observed when grown on inert PLL, particularly upon inclusion of sulfatide in the plasma membrane (Figure 4D). Moreover, this shift was reflected by a substantial decrease in the lateral diffusion rate of the protein relative to that measured in the control cells (OLN-P) (Figure 4D and E). Accordingly, we hypothesized that the differences observed in the s-FCS measurements for OLN-GS cells on Fn versus Ln2 might also be due to differences in the microdomain association of PLP. To investigate this possibility, OLN-P and OLN-GS cells cultured on either Fn or Ln2 were extracted with CHAPS followed by OptiPrep density gradient analysis. The data, illustrated in Figure 8A and B, demonstrate that in cells grown on Ln2, the fraction of CHAPS-insoluble PLP was significantly higher in OLN-GS cells (approx. 70%) than in control OLN-P cells (approx. 45%). In contrast, cells cultured on Fn showed reduced partitioning of PLP into CHAPS-insoluble domains to approx. 30%. Moreover, we did not detect significant differences in the CHAPS-insoluble domain association of PLP in OLN-GS versus OLN-P cells (Figure 8B). Clearly, the enhanced partitioning of PLP into CHAPS-insoluble microdomains in GS-expressing cells, grown on Ln2, is also reflected by a strongly reduced lateral diffusion rate of the protein in these cells compared to the rate observed in OLN-P cells. Intriguingly, such a correlation is not apparent in cells grown on Fn, suggesting that factors other than a restriction in lateral diffusion due to raft localization diminish PLP dynamics in these cells (see Discussion).

## Discussion

In this study, we have examined by means of biochemical and biophysical tools how the myelin typical galactolipids GalC and sulfatide affect the organization, distribution and dynamic properties of myelin-specific proteins MBP and PLP in the plasma membrane of OLN-93 cells in conjunction with a modulatory effect of the ECM. When exposed to an inert substrate PLL or to the physiologically relevant and myelination-promoting ECM protein Ln2, we show that the presence of sulfatide altered PLP′s membrane microdomain association and mobility. In contrast, when grown on the myelination-inhibiting substrate Fn, the membrane microdomain association of PLP is disturbed in the presence of sulfatide and the diffusion rate of the protein increased. Interestingly, while PLP preferentially associates with sulfatide-containing membrane domains, MBP dynamics is mainly governed by GalC, its presence causing 18.5-kDa MBP, as a peripheral membrane protein, to (partly) associate with detergent-insoluble microdomains. Given the asymmetric distribution of MBP and GalC, this effect presumably relies on a transmembrane GalC-mediated stimulus.

Myelin galactolipids GalC and sulfatide are crucial for maintaining myelin integrity as is apparent from knockout experiments of enzymes responsible for their synthesis. In the absence of galactolipids, PLP expression remains unaltered however the protein no longer integrates within CHAPS-resistant microdomains [36], [49], [50]. Furthermore, in these GalC-knockout models, down regulation of the expression of several proteins was reported, e.g., members of the septin family and SIRT2, which were also down regulated in a PLP-null mouse model [51]. This might suggest that the absence of galactolipids may create a similar environment, in which PLP is malfunctioning. One possibility is that PLP may only function properly by localizing in the correct plasma membrane microdomain, which might directly or indirectly relate to proper functioning of other proteins as well. However, current knowledge of how membrane microdomain association of PLP, and involvement of distinct galactolipids in this process, regulate its dynamic properties, is scanty. To investigate this possibility, we employed the oligodendrocyte cell line OLN-93 [32] in which we selectively (over)expressed GalC or GalC and sulfatide, conditions that are very difficult to mimic with primary cells or myelin tissue. Due to the heterogeneous cellular distribution of PLP, localizing as a transmembrane protein in both highly dynamic cytoplasmic transport vesicles and the plasma membrane, we applied circular s-FCS, a more advanced technique that can provide more consistent results than point FCS in model membranes, such as GUVs (see [46]). As shown here, we successfully applied this approach to living cells by adjusting the measurement and analysis conditions such that for the first time the signal contribution from the protein associated with the cytoplasmic vesicles is minimized while that of the plasma membrane is enhanced. Moreover, we demonstrate that PLP preferentially co-localizes with sulfatide rather than GalC, at the plasma membrane. As a consequence, PLP, which is CHAPS-soluble in OLN-93 cells devoid of galactolipids, is mainly recovered in CHAPS-insoluble fractions on inert PLL, suggesting its recruitment in detergent-insoluble sulfatide-enriched microdomains. Consistent with its capture in restricted domains, PLP′s lateral diffusion rate as determined by s-FCS was slowed down in the presence of sulfatide, as compared to its dynamics in galactolipid-deficient OLN-93 cells and OLN-93 cells that only express GalC.

Remarkably, although 18.5-kDa MBP is a peripheral membrane protein, its dynamics are affected by the presence of galactolipids, in particular GalC. Thus, 18.5-kDa MBP co-distributed to a major extent with GalC in spite of their asymmetric membrane localization. Moreover, in GalC-expressing cells, a substantial fraction (∼ 30%) of the protein was recovered in CHAPS-insoluble microdomains. This effect was absent in galactolipid deficient cells and, surprisingly, in cells expressing both GalC and sulfatide. Intermixing of sulfatide with GalC pools may repress a specific transmembrane-induced GalC effect on MBP distribution in the inner leaflet and, therefore, it is tempting to suggest that GalC may function as a cell surface transmitter, propagating the signal that triggers MBP′s peripheral association with microdomains. Indeed, GalC-containing liposomes or glycol-nanoparticles containing GalC/sulfatide, when added to primary OLGs caused a redistribution of MBP at the cytoplasmic surface and GalC at the extracellular surface together with a redistribution of some phosphorylated proteins involved in signal transduction [52]. It is also possible that the presence of GalC in the outer leaflet may change the inner leaflet lipid organization, which might lead to a change in MBP′s phosphorylation status. It has been shown, for example, that phosphorylated MBP associates with CHAPS-insoluble domains in bovine myelin [51], [52] and addition of anti-GalC antibodies caused a decrease in phosphorylation of MBP [53]. Remarkably, in spite of an enhanced association with detergent-resistant domains in GalC-expressing cells, *z*-scan point FCS and RICS analysis revealed that the mobility of 18.5-kDa MBP was significantly higher in OLN-G cells compared to OLN-GS. The apparent difference in raft association between the peripheral protein MBP (association) and the transmembrane protein PLP (integration) is also reflected by the much slower diffusion rate seen for PLP than for 18.5-kDa MBP in the presence of GalC. Differences in the mode of protein interaction with microdomains have been reported before to give rise to differences in lateral diffusion coefficients [44]. However, it should be noted that it is currently unclear to what extent the various MBP pools (CHAPS-soluble or -insoluble) contribute to the analyses of the lateral diffusion coefficients. In addition, it is probable that the raft association of MBP is not the only factor determining MBP′s diffusion. In fact, the inner leaflet contains fewer barriers to protein diffusion than the outer leaflet, which also depends on membrane anchorage [54]. Furthermore, the presence of only GalC might alter the interaction partners of MBP and thereby the mobility of the protein. In this context it is relevant to note that we have observed in primary OLGs by extraction *in situ* that GalC-positive microdomains are largely confined to the myelin membranes, whereas sulfatide containing microdomains are restricted to the cell body and primary processes (unpublished observations). Further investigations will be necessary to understand the role of GalC- and/or sulfatide-enriched microdomains in MBP dynamics in myelin membranes, and may benefit from the application of artificial membranes such as GUVs and Giant Plasma Membrane Spheres [55], [56] to more carefully define the distinct interactions that determine the diffusion coefficients, including the possibility to create solely sulfatide-containing domains.

It is becoming increasingly apparent that changes in the ECM affect myelination efficiency and alterations in the lateral organization of myelin proteins have been proposed to be part of the underlying mechanism [13], [20], [48], [57], [58]. Specifically, at physiological conditions, axonal Ln2 [59] promotes (re)myelination whereas, at pathological conditions such as MS lesions, Fn inhibits (re)myelination [48]. The present approach, which allows for carefully controlled expression of galactolipids, in conjunction with careful biophysical measurements of the lateral dynamics of PLP and MBP thus provided the unique opportunity to selectively investigate the role of the distinct galactolipids and myelin protein dynamics in an ECM-dependent manner. Our present findings demonstrate that Ln2 and Fn did not affect MBP′s dynamics. Apparently, as shown in a recent study secreted neuronal signals rather than the ECM might be crucial for the lateral organization of MBP as such signals can shift the localization of MBP completely to CHAPS-insoluble fractions [13]. In contrast, the dynamics and lateral membrane organization of PLP are dependent on the ECM which the cells encounter. Thus, on Fn, PLP resides mainly in CHAPS-soluble microdomains whereas on Ln2, i.e., at physiological conditions, PLP redistributes from CHAPS-soluble to CHAPS-insoluble domains. This observation is very reminiscent of a previous study, demonstrating that in cultured primary OLGs raft association of the myelin protein NF155 is decreased in the presence of Fn, which is detrimental to (re)myelination [37], [60]. In fact, recent work from our laboratory has demonstrated a decrease in the partion of sulfatide into detergent-insoluble domains when primary OLGs are cultured on Fn [61], suggesting a severe disruption of membrane microdomain assembly. Consistent with these observations, the extent of PLP-sulfatide co-distribution at the plasma membrane decreased 3-fold in sulfatide-expressing OLN-GS cells when the cells were cultured on Fn rather than Ln2 (Figure 7C). Furthermore, the lateral diffusion rate of PLP in OLN-GS cells cultured on Fn is relatively high compared to measurements on Ln2. Remarkably, we also observed that the diffusion rate of PLP in the plasma membrane of control OLN-P cells, i.e., in cells not expressing galactolipids, was three fold slower when the cells were grown on Fn, rather than Ln2. Since this difference is not reflected by similar differences in CHAPS-insolubility (Figure 8A and B), these data suggest that Fn apparently displays a rigidifying effect on the cell surface, which restricts lateral (protein) mobility. Evidently, such an effect may obviously frustrate membrane dynamics necessary for cellular trafficking including myelin biogenesis [20], thus potentially rationalizing the detrimental effect of Fn on (re)myelination. However, additional work will be required to clarify the underlying mechanism.
